# Bone Marrow Adipocyte: An Intimate Partner With Tumor Cells in Bone Metastasis

**DOI:** 10.3389/fendo.2018.00339

**Published:** 2018-06-22

**Authors:** Guojing Luo, Yuedong He, Xijie Yu

**Affiliations:** ^1^Laboratory of Endocrinology and Metabolism, Department of Endocrinology and Metabolism, State Key Laboratory of Biotherapy, West China Hospital, Sichuan University, Chengdu, China; ^2^Department of Gynecology, West China Second University Hospital, Sichuan University, Chengdu, China

**Keywords:** bone marrow adipocyte, adipose, bone metastasis, interleukin-6, tumor necrosis factor-α, leptin, adiponectin

## Abstract

The high incidences of bone metastasis in patients with breast cancer, prostate cancer and lung cancer still remains a puzzling issue. The “seeds and soil” hypothesis suggested that bone marrow (soil) may provide a favorable “niche” for tumor cells (seed). When seeking for effective ways to prevent and treat tumor bone metastasis, most researchers focus on tumor cells (seed) but not the bone marrow microenvironment (soil). In reality, only a fraction of circulating tumor cells (CTCs) could survive and colonize in bone. Thus, the bone marrow microenvironment could ultimately determine the fate of tumor cells that have migrated to bone. Bone marrow adipocytes (BMAs) are abundant in the bone marrow microenvironment. Mounting evidence suggests that BMAs may play a dominant role in bone metastasis. BMAs could directly provide energy for tumor cells, enhance the tumor cell proliferation, and resistance to chemotherapy and radiotherapy. BMAs are also known for releasing some inflammatory factors and adipocytokines to promote or inhibit bone metastasis. In this review, we made a comprehensive summary for the interaction between BMAs and bone metastasis. More importantly, we discussed the potentially promising methods for the prevention and treatment of bone metastasis. Genetic disruption and pharmaceutical inhibition may be effective in inhibiting the formation and pro-tumor functions of BMAs.

## Introduction

Bone is a common site of distant metastasis in breast cancer, prostate cancer and lung cancer (1). Once bone metastasis occurred, the prognosis is poor, especially for lung cancer patients. However, the mechanism of bone metastasis is still elusive until now. Paget's “seeds and soil” hypothesis is widely used to explain this phenomenon (2). Bone may provide a comfortable “niche” (soil) for tumor cells (seed) and help them survive and then invade the bone. Numerous studies have demonstrated that certain genes and cytokines are associated with bone metastasis. Most of these factors are adhesive factors, inflammation factors and chemotactic factors (3). However, majority of these factors are related to “seed” but not “soil”. In reality, suitable “soil” may determine the destiny of “seed”, as < 0.01% of CTCs can ultimately form a distant metastasis (4).

Bone marrow contains a great variety of different cells, for example, red blood cells (RBCs), white blood cells (WBCs), platelet, endothelial cells, pericapillary cells, BMAs, etc. All these cells could constitute fertile “soil” and provide a suitable environment for tumor cells. Among them, BMAs are most abundant in bone marrow cavity. They constitute about 70% of bone marrow volume in adults (5). However, the important roles of BMAs have been overlooked for a long time. BMAs were previously regarded as a passive “filler” (6). Recently, more and more researchers try to unravel the long-neglected roles of BMAs. Like the white adipose tissue (WAT), bone marrow adipose tissue is a highly active endocrine organ (7). Besides, similar to the heterogeneity between WAT depots (8), the variation of region distribution and functions of BMAs were demonstrated in rat, mouse and human. The distal “constitutive BMAs” (cBMAs) are much abundant and remain stable in cold environment. In contrast, the proximal “regulated BMAs” (rBMAs) are much active and could disappear in long-term low temperature (9). In addition to bone metabolism, BMAs are involved in energy balance and bone metastasis. BMAs could secrete a variety of adipokines (adiponectin and leptin, etc.) and inflammatory factors (interleukin-6 and tumor necrosis factor-α, etc.). Besides, BMAs could be the energy source of tumor cells and could be sensitive to the cytokines released by tumor cells. Free fatty acid (FFA) released by BMAs could be utilized by tumor cells. In this way, BMAs could support tumor cells to adhere, colonize and invade bone tissue in bone marrow microenvironment. In this review, we summarize our current understanding of the relationship between tumor cells and BMAs. In addition, we discuss the new therapeutic possibilities for the treatment of bone metastasis.

## Different roles of WAT and BMAs in the tumorigenesis and bone metastasis

The association of WAT and tumor risk was initially found in epidemiological data. Obesity, which is characterized by excessive accumulation of WAT, increases the tumor risk of some cancers (10). Most of these obesity-related cancers are located within the digestive system, for example, esophageal adenocarcinoma, gastric cardiac cancer, colon cancer, rectum cancer, cancer of biliary tract system, and pancreas cancer. Obesity also increases the risk of cancers in the urogenital system, including breast cancer, endometrium cancer, ovary cancer, and kidney cancer (11). What is more, obesity is also associated with an increased risk for multiple myeloma (MM) (12).

Decades of research demonstrated that obesity could promote cancer progression. However, we currently have little knowledge of the relationship between WAT and bone metastasis. Some obesity-associated cancers are surrounded by WAT and grow in the adipose tissue abundant environment. For example, breast cancer and kidney cancer usually colonize in adipose tissue enrich niches, and some other types of cancers, such as colon cancer and ovary cancer, are always adjacent to the abdominal fat. These histological characteristics are yet another type of evidence supporting the relationship between WAT and cancers. In addition, obesity-associated cancers have distinct incidences of bone metastasis. Breast cancer preferentially metastasizes to bone. 65–75% of breast cancer patients develop bone metastases (13). In contrast, there is low probability of bone metastasis in other obesity-related cancers, especially in digestive cancers. The incidence of skeleton metastasis is as low as 4–6% in colon cancer patients (14). More importantly, colon cancer with skeleton metastasis without other organs involvement is rare (15). Both prostate cancer and lung cancer patients have high incidences of bone metastasis. However, the relationship between obesity and prostate cancer or lung cancer is still not conclusive. The research results of prostate cancer risk and obesity are in conflict. Some studies demonstrated the positive correlation between obesity and prostate cancer risk (16, 17). Interestingly, another study demonstrated that obesity increased the risk of high-grade prostate cancer but decreased the risk of low-grade prostate cancer (18). However, a recent review stated, there was no association between obesity and prostate cancer risk (19). As for lung cancer, the results are quite astonishing. Obesity even reduced the lung cancer risk of the current and former smokers (20). More importantly, obesity did not increase the incidence of bone metastasis in breast cancer patients (21). These findings at least indicate that high frequencies of bone metastasis in breast cancer, prostate cancer, and lung cancer is not necessarily correlated with WAT. Although WAT has the potential of enhancing the invasion capacity of cancer cells, little is known about its specific roles in bone metastasis.

It is known that BMAs share many similarities with WAT. Many genes associated with adipogenesis and fat metabolism including peroxisome proliferator activated receptor gamma (PPAR-γ), CCAAT/enhancer binding protein α (C/EBPα), perilipin and hormone-sensitive lipase (HSL) are comparably expressed in WAT and BMAs (22). Recent studies have demonstrated that BMAs are different from WAT in several ways. Firstly, their origin is different. BMAs are mainly derived from Sca1^+^CD45^−^CD31^−^ (23) or LepR^+^CD45^−^CD31^−^ bone marrow mesenchymal cells (BMSCs) (24). In contrast, WAT is mainly derived from CD34^+^ CD44^+^ CD45^−^ CD31^−^ CD19^−^ adipose tissue stem cells (ADSCs) (25), which reside in the white adipose vascular niche (26). Secondly, their endocrine functions are different. BMAs could secrete more adiponectin than WAT during caloric restriction (7) and aging (27). The expression of leptin in BMAs is in conflict in different studies (27, 28). More importantly, BMAs are located near the endosteal surface of the diaphysis, as well as the trabecular bone of the epiphysis and metaphysis. The latter is the predilection site of bone metastasis, which is the site of active bone remodeling. Their adjacent relationship could indicate the potential relationship in bone metastasis. In addition, considering the abundance of BMAs in bone marrow, accumulating evidence suggests that BMAs are crucial in the development of hematological malignancies. BMAs directly interact with hematologic tumor cells in the bone marrow and could promote tumor cells to grow and survive in MM (29, 30) and leukemia model (31). In brief, the roles of BMAs in bone destruction and bone metastasis have been in the spotlight in recent years.

## BMAs provide energy for tumor cells

Glucose is a well-known dispensable source to support tumor growth. Besides, FFA, which is derived from sequential hydrolysis of stored triacylglycerol (32), is another important substrate for tumor metabolism. FFA could turn into lipid droplets in the cytoplasm of tumor cells. These lipid droplets are capable of increasing the survival rate of tumor cells. High fat content in some tumor cells may distinguish them from non-malignant cells. The human lung cancer cell line A549 contained more than 10 times the amount of lipid droplets than the normal lung fibroblast cell line WI38 (33). Similar conclusion could be found in the human breast cancer cell line MDA-MB-231 (34), which is often used in animal model of bone metastasis. Small lipid droplets were also observed in the myeloma cells when they were co-cultured with BMAs (35). In regard to the important roles of lipid metabolism in tumor cells, researchers have tried to improve therapeutic effects by inhibiting the accumulation of lipid droplets in tumor cells. *De novo* fatty acid synthesis is performed via glycolysis and glutaminolysis in normal condition (36). The rate-limiting enzyme in *de novo* fatty acid synthesis is acetyl-CoA carboxylase (ACC), which can be inhibited by ND-646. ND-646 treatment resulted in the loss of neutral lipids and a 90% reduction in total fatty acid content in non-small-cell lung cancer (NSCLC) cells, including the predominant saturated fatty acids palmitate and stearate. More importantly, the proliferation of tumor cells was also inhibited by ND-646 (37). However, in some tumors that are not inclined to metastasize to bone, the results are different. Marin and colleagues found that liver-specific knockout of ACC resulted in increased cell vitality and greater tumor incidence in mice treated with carcinogens diethylnitrosamine (DEN) (38). In addition, the excessive accumulation of lipid droplets in tumor cells does not always exert a beneficial effect. CD36, a cell surface scavenger receptor, is mainly responsible for the fatty acid transportation. Once CD36 was inhibited by CD36-neutralizing antibodies, large lipid-abundant tumor cells would appear, as well as a significantly reduced incidence of metastasis (39). From this viewpoint, the proper amount of neutral fat in tumor cells may be needed for their rapid proliferation, especially for the tumor cells that preferentially metastasize to bone.

Some tissues and organs also utilize FFA from adjacent adipocytes in normal physiological conditions. For example, epithelial cells within mouse mammary gland could induce the lipolysis of neighboring adipocytes to make use of the FFA during lactation (40). Thus, it is not surprising that tumor cells also possess this inherent capacity, most prominently, breast cancer cells. In addition to *de novo* fatty acid synthesis, cancer cells could directly acquire FFA from adipocytes. This additional source of fatty acid is extraordinary important for tumor cells in an energy deprivation state. In co-culture condition, fatty acid released from adipocyte could be transferred to colon cancer cells (41). This amazing phenomenon was confirmed by fluorescent microscope *in vitro*. An *in vivo* experiment also supported this finding. Wen and his colleagues demonstrated that tumor growth can be significantly enhanced if SW480 cells were mixed with adipocytes before they were injected into mice. One month later, adipocytes were no longer present in the tumor sections. They speculated that these mature adipocytes fueled the adjoining cancer cells and consumed themselves during tumor progression (41). Another *in vivo* experiment may support this hypothesis. Wang and colleagues found that the number of unilocular and multilocular BMAs increased significantly in the bone metastasis niche during the first week. However, a notable reduction of BMAs was observed after 2 weeks. Further studies demonstrated that the increase of BMAs at the early stage of bone metastasis resulted from the enhanced adipogenic differentiation of preadipocytes under the boost of melanoma cell-derived factors (42). But as the tumor proliferated rapidly, melanoma cell enhanced the dedifferentiation of mature adipocyte: from lipid-droplet abundant adipocytes to fatless fibroblasts. Delipidation of mature adipocytes was accompanied with the decreased expression of adipocytes markers, including CCAAT/enhancer binding protein beta (C/EBP-β), PPAR-γ, fatty acid binding protein 4 (FABP-4) and leptin (42). These findings may indicate that tumor cells promote the BMA differentiation during the early stage. In later stages, tumor cells begin to stimulate the dedifferentiation of mature BMAs to satisfy their increasing energy requirement.

**Figure 1:**
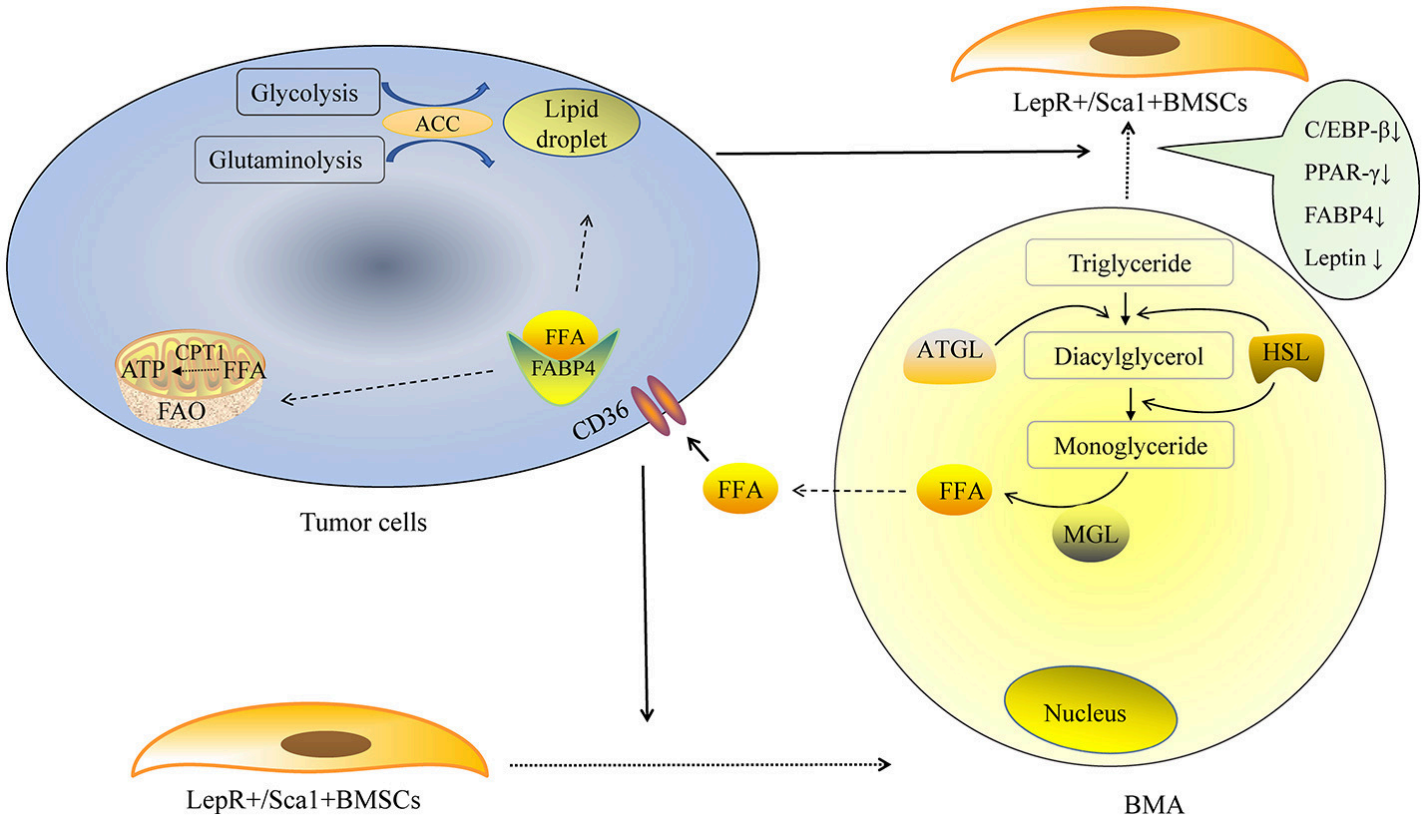
BMAs provide energy for tumor cells. *De novo* fatty acid synthesis is performed via glycolysis and glutaminolysis in tumor cells. The rate-limiting enzyme ACC is indispensable in the lipid droplet formation in tumor cells. Exogenous FFA could derived from BMAs. Tumor cells secrete some factors to promote the adipogenic differentiation of LepR^+^/Sca1^+^BMSCs in the early stage, as the tumor proliferate rapidly, large amount of tumor cells promote mature BMAs to de-differentiate into fatless fibroblast in the later stage. Lipid-breakdown factors from tumor cells enhance the lipolysis of mature adipocytes. The initial step of triglyceride hydrolysis is predominantly performed by ATGL and triglyceride is broken up to diacylglycerol. Then diacylglycerol is hydrolyzed into monoglyceride. FFA is transported by fatty acid binding protein 4 (FABP4). Tumor cells could also express FABP4 that could transfer free fatty acid when they are co-cultured with BMAs. Tumor cells transport FFA from cytosol into mitochondria for fatty acid oxidation (FAO) and produce ATP. This process is mainly performed by carnitine palmitoyltransferase-1 (CPT1A).

The mechanism of lipolysis in BMAs can also be explained by some recent studies. One of them is the region-specific variation of BMAs. In 2015, Scheller and colleagues made an important discovery in their pioneering study, in which they defined “regulated bone marrow adipocytes” (rBMAs) and “constitutive bone marrow adipocytes” (cBMAs) for the first time (9). “Regulated” means “changeable.” rBMAs are located primarily in the proximal long bones, active sites of hematopoiesis. rBMAs accumulate in the aging process and decrease remarkably in cold environments. In contrast, cBMAs fill the cavities of the distal long bones and appear earlier. cBMAs have relatively larger sizes compared to rBMAs. More importantly, cBMAs remain stable despite challenging conditions. These new findings may partially explain the predilection sites of bone metastasis. In general, tumor cells prefer to metastasize to rBMAs-enriched regions (proximal femur, hip, and lumbar spine, etc.) that contain smaller and less stable adipocytes. Coincidentally, rBMAs but not cBMAs would disappear after 3 weeks cold exposure. This phenomenon may be similar to the disappearance of BMAs in bone metastasis sites. The second is the pathogenesis of cachexia. Cachexia, which is characterized by wasting WAT and muscle, is a well-known debilitating complication in terminal cancer patients (43). Lipid-breakdown factors from cancer cells enhance the lipolysis of mature adipocytes. The mobilization of triglyceride stored in the lipid droplets requires lipolysis of triglyceride. This process is mainly mediated by adipose triglyceride lipase (ATGL) and HSL (44). The initial step of triglyceride hydrolysis is predominantly performed by ATGL. Triglyceride is initially broken up into diacylglycerol then hydrolyzed into monoglyceride. FFA is transported by FABP4, which is predominantly expressed in adipocytes. Strong expression of FABP4 could also be detected in prostate cancer with skeleton metastasis (45). In addition, a significant upregulation of FABP4 was observed in ovarian, colon, and breast cancer cell lines co-cultured with WAT (36). The most obvious change was detected in breast cancer cell lines T47D, with an increase around 70-fold. Leukemia cells could also express FABP4 in order to transfer FFA when they were co-cultured with BMAs (46). Tumor cells could transport FFA from cytosol into mitochondria for fatty acid oxidation (FAO) and produce ATP. This process is mainly performed by carnitine palmitoyltransferase-1 (CPT1A). The expression of CPT1A in acute myelocytic leukemia (AML) cells co-cultured with BMAs was significantly up-regulated; knockdown of CPT1A could reduce the survival and proliferation of AML cells when co-cultured with BMAs (46). It seems that fatty acid oxidation (FAO) is vital for the use of FFA in tumor cells and could be a target to inhibit tumor growth. We suppose that, BMAs are the important source of energy for tumors residing in the bone marrow niche (Figure 1). The lipid-mobilizing factors may be more prevalent in the bone marrow microenvironment in which tumor cells directly contact with BMAs.

## The enhancement of tumor cell proliferation and resistance to chemotherapy and radiotherapy by BMAs

Adipocytes provide more than just energy for tumor cells. As early as 1992, Elliott and colleagues found that co-transplantation of breast cancer cells with adipose tissue led to increased tumor growth (47). In addition, co-culture with adipocytes increased the invasive capacity not only in the metastatic breast tumor cells line 4T1, but also in the non-metastatic cell line 67NR (48). A further study identified salt-inducible kinase 2 (SIK2) as a mediator between tumor cells and adipocytes. Mature adipocytes enhanced intracellular calcium ions in tumor cells and induced the auto phosphorylation of SIK2, which then inhibited ACC and activated phosphoinositol 3 kinase–protein kinase B (PI3K/AKT) signaling (49). A more recent study showed that gonadal adipose tissue provided the assistance for leukemia stem cells (LSCs) to escape from chemotherapy, and thus prompted the recurrence of leukemias (50). The above studies show the important roles of WAT in tumor progression. Similarly, BMAs also play a crucial role in tumor development. Rapid expansion (9–32%) of BMA was observed in tumor patients over 1 year (51), far more than the averaging 1% per year in the natural process of aging (52). Moreover, chemotherapy and radiotherapy could further increase the number of BMAs, especially in the sacral vertebrae (7). The increased BMAs assist tumor cells in the process of tumor evasion and bone destruction after irradiation and chemotherapy. Leukemia and MM are ideal disease models to explore the relationship between BMAs and cancer cells. These malignant cells reside in the bone marrow niche and directly contact with BMAs. The proliferation of leukemia cells was increased when co-cultured with BMAs. As the chemotherapy drug vincristine was added to the co-culture system, BMAs obviously inhibited the anti-tumor effects of vincristine. BMAs protected leukemia cells from chemotherapy-induced apoptosis by overexpressing growth signals B-cell lymphoma-2 (Bcl-2) and the proviral integration site for moloney murine leukemia virus 2 (Pim2) (53). In the MM model treated with melphalan, the result is similar. Human BMAs activated autophagy and inhibited caspase cleavage and apoptosis via Janus kinase 2/signal transducer and activator of transcription 3 (Jak/STAT3) signaling (54). Another study involving leukemia cells showed that BMAs activated AMP activated protein kinase (AMPK)-dependent cytoprotective autophagy and downregulated Akt/mechanistic target of rapamycin (mTOR) signaling in the starvation condition (31).

**Figure 2:**
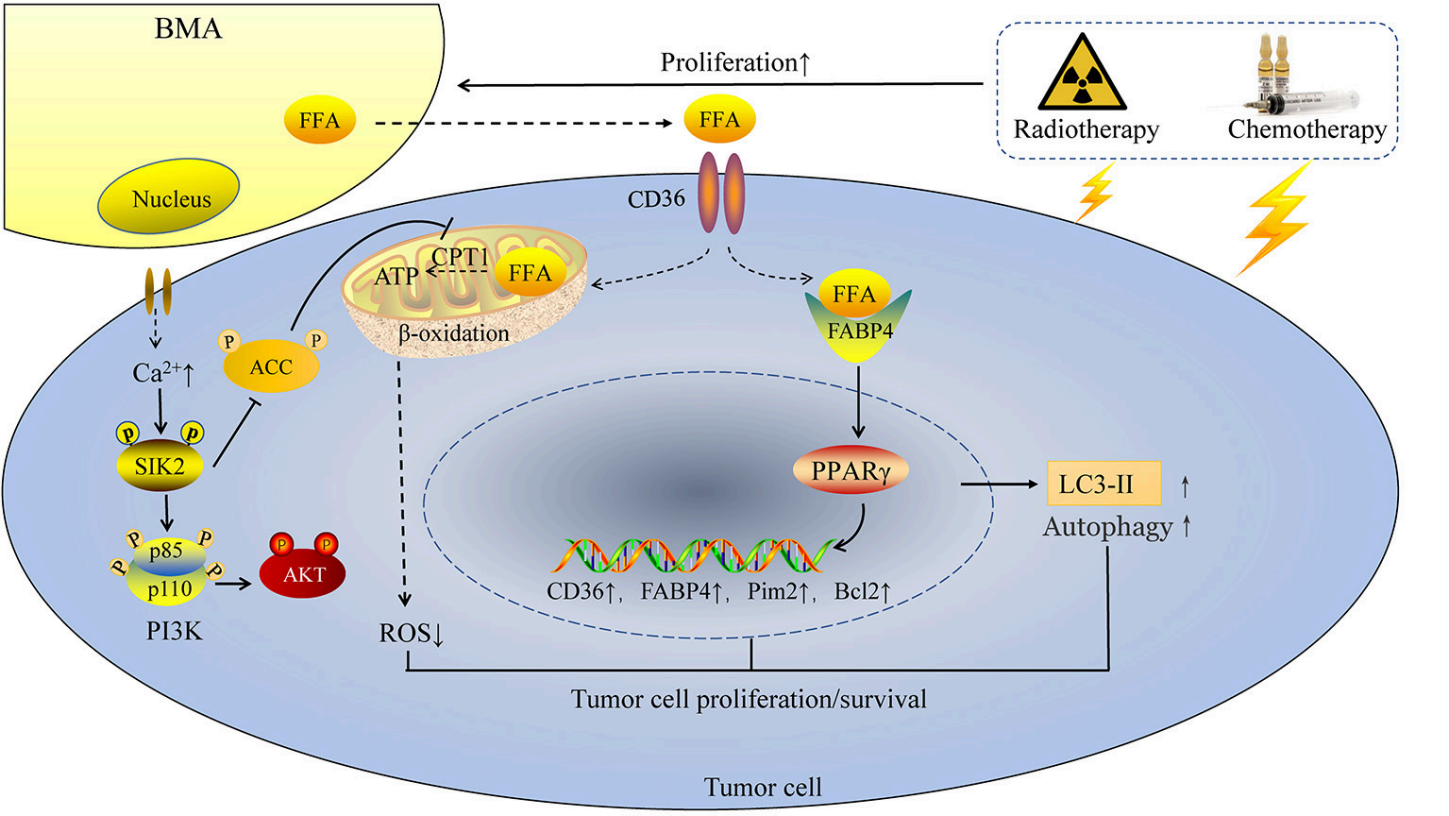
BMAs enhance tumor cell proliferation and resistance to chemotherapy and radiotherapy. Chemotherapy and radiotherapy enhance the rapid expansion of BMAs in tumor patients, followed by enhanced intracellular calcium ions in tumor cells and increasing FFA released from BMAs. On one hand, FFA is transported into tumor cells by CD36 and then FFA is transported to the nucleus by FABP4. FFA binds to the essential ligands of nuclear receptor PPARγ, followed by enhanced expression of CD36, FABP4, Pim2 and Bcl2, as well as the autophagy marker LC3-II. On the other hand, BMAs induce the autophosphorylation of SIK2, which then inhibits ACC and activates PI3K/AKT signaling. Increasing FFA from BMAs is transported into mitochondria and produces ATP in the energy deficiency condition. In this way, ROS is reduced and tumor cells survival in the bone marrow niche is enhanced.

In tumors with high frequency of bone metastasis, BMAs exert its pro-tumor effects mainly by modulating lipid metabolism associated genes, including FABP4, PPARγ and CD36. Abundant amounts of FABP4 was found present in bone metastasis samples from mice and human; BMAs promoted prostate cancer growth dependent on FABP4 (45), which was activated by PPARγ (55). The mechanism is partly mediated by c-Jun N-terminal kinase (JNK)/MAPK pathways (56). Fatty acid transporter CD36 is also indispensable in the process of tumor metastasis. Recent studies demonstrated that CD36 is a marker of metastasis-initiating cells (39). In the FFA enriched environment, CD36 is also important for the occurrence of bone metastasis. Breast cancer and prostate cancer cells co-cultured with BMAs showed a great upregulation of CD36 (45). BMAs release FFA, FFA is then transported into cytoplasm via CD36. Next, FFA is transported to the nucleus by FABP4. FFA binds to the essential ligands of nuclear receptor PPARγ, leading to upregulation of anti-apoptotic factor Bcl2 (31) (Figure 2).

The mechanism responsible for the large increase in BMAs in tumor development could be partly associated with the awakened BMA progenitors. Previous studies showed that quiescent BMA progenitors would enter into cycle (S/G2/M) after irradiation treatment (57). Bone marrow cells secreted large quantities of bone morphogenetic protein-4 (BMP4) in response to irradiation, which promoted the adipogenic differentiation of BMSCs (58). Among the various adipocytokines, leptin is the key factor that have been shown to implicated in the resistance to chemotherapy and radiotherapy (59, 60). Leptin could activate the PI3K/Akt and mitogen-activated protein kinase (MEK)/extracellular-regulated protein kinase (ERK) signaling pathway, and consequently promote cell proliferation and cell survival (59). This provides a potential therapeutic avenue for bone metastasis. Inhibiting the adipogenic differentiation of BMSCs may be useful to block these pro-tumor effects. However, a recent research revealed that the increase of BMAs inhibited the survival of AML cells (61). This new conclusion is vastly different from any other researches and may require further verification.

## The endocrine and paracrine functions of BMAs in bone metastasis

The endocrine and paracrine functions of BMAs are different from WAT. So far, numerous studies have demonstrated these differences. The adipokines and cytokines released from BMAs are important factors in bone metastasis. Among these factors, leptin and adiponectin are two major adipokines released by adipocytes. Surprisingly, the effects of these two adipokines on tumor metastasis generally contradict each other.

### The effect of leptin on tumor cells and bone metastasis

Leptin is a 16 kDa multifunctional protein released by adipocytes, including WAT and bone marrow fat (62). Early researches focused on its functions on appetite and energy balance. But its functions are more than that. Leptin exerts its effects by binding to its receptor leptin receptor (LepR). Unlike other hormones, leptin binds to its central and peripheral LepR and produces different even opposite effects on bone formation (63). Binding of leptin to its central receptors could induce bone loss, whereas binding of leptin to its peripheral receptors could increase bone mass. In addition to its roles in energy balance and bone metabolism, leptin may also play an important role in bone metastasis. First, leptin may enhance the formation of BMAs. Mature adipocytes release leptin, which could bind to LepR on BMSCs and promote the adipogenesis of BMSCs (57). More importantly, LepR(+) BMSCs are the main source of BMAs (64). In the primary culture of human BMAs, BMAs could secrete more amounts of leptin than WAT (28). The increased leptin could bind to LepR and activate Jak2/STAT3 signaling in BMSCs, thus forming more BMAs (24) and creating a positive feedback for tumor cells. Second, leptin/LepR pathway could promote the oncogenesis, proliferation and colonization of tumor cells in the bone marrow niche. Leptin attracted the breast tumor cells to migrate to bone marrow niche (65). Adipocytes also enhanced the proliferative effect of colon cancer cells. However, this effect was leptin dependent. The adipocytes from leptin-deficient ob/ob mice showed no enhanced proliferation effect on tumor cells unless exogenous leptin was administrated. However, the pro-tumor effects of leptin depend on its receptor LepR, which is expressed in various tumors, including breast cancer, prostate cancer, and colon cancer (66–68). Leptin activated the downstream Jak2/STAT3 signaling in LepR(+) tumor cells, which led to enhanced proliferation, survival and migration of tumor cells (69). Despite of the elevated serum level of leptin, LepR-deficient mice (db/db) failed to develop breast cancer in an oncogene-induced rodent model (MMTV-TGF-alpha mice) (70). Leptin also enhanced the metastasis of human lung cancer cell line A549 by activating TGF-β signaling (71). A few studies, however, showed conflicting results, for instance, Samuel-Mendelsohn and colleagues demonstrated that leptin promoted the apoptosis of human prostate cell line (72).

Sex steroids play an indispensable role in the pro-tumor effect of leptin. The unbalance of sex hormones and their receptors may increase the risk of bone metastasis. Breast cancer and prostate cancer are referred to as sex hormone-dependent cancers (73). More remarkably, breast cancer and prostate cancer patients have the highest frequency of bone metastasis. This phenomenon may indicate that estrogen/estrogen receptor (E/ER) and androgen/androgen receptor (A/AR) signaling are extremely important in bone metastasis. An *in vitro* experiment showed that mature adipocytes promoted the proliferation of human and rodent ER (+) breast tumor cell lines, including MCF-7, ZR75-1, T47-D, and SPI cells, instead of ER (-) tumor cell line MMT 060562 cells (47, 74). The effect of leptin on tumor growth is dependent on A/AR or E/ER signaling. Leptin administration obviously enhanced the proliferative capacity of androgen-dependent prostate cell line LNCaP cells, but this effect did not appear in androgen-independent prostate cell lines PC3 and Du-145. On the other hand, exposure to leptin led to a significant reduction in migration and invasion of androgen-insensitive prostate cells by activation of MAPK/STAT3 pathway (75). However, the results from other studies are conflicting. Adipocyte derived leptin promoted the proliferation of androgen-independent human prostate cancer cell lines DU145 and PC-3 rather than androgen-dependent LNCaP-FGC cells, although both cell types expressed functional LepR isoforms (76, 77). These contradictory results reveal the complicated network between different tumors and the bone marrow environment. Even the non-hormone dependent cancers, such as lung cancer, could be associated with sex hormone in bone metastasis. Human small cell lung cancer cell lines SBC-5, which is apt to metastasize to bone, expressed AR and ERβ, but not ERα; by contrast, human small cell lung cancer cell line SBC-3, which does not metastasize to bone, expressed ERα and ERβ, but not AR (78). The inhibition of androgen by castration or androgen receptor antagonist in male mice resulted in a much higher frequency of bone metastasis in small cell lung cancer mice (78). These studies may indicate the important roles of AR in bone metastasis of small cell lung cancer. On the other hand, androgen deprivation therapy would increase bone marrow fat (79), leading to a potentially higher risk of bone metastasis.

### The effect of adiponectin on tumor cells and bone metastasis

Adiponectin is a 30 kDa secreted protein with multiple functions and is exclusively released by adipocytes, including WAT and BMAs (80). Adiponectin plays its roles by binding to its receptors. In general, adiponectin could bind to two receptors, adiponectin receptor 1 (AdipoR1) and adiponectin receptor 2 (AdipoR2). Full-length adiponectin binds preferentially to AdipoR2 (81), whereas proteolytic cleavage production of adiponectin (gAcrp30) is more likely to bind with AdipoR1 (80).

Previous studies showed that WAT was the main sources of adiponectin (82). Surprisingly, recent studies reported that BMAs could produce more adiponectin than WAT, especially in tumor patients. The increase of BMAs in tumor patients at 6 and 12 months after chemotherapy or radiotherapy treatment contributed to the higher serum level of adiponectin from BMAs but not WAT (7). Consistent with the increase number of BMAs and serum level of adiponectin, AdipoR1 expression in leukemia cells also increased when co-cultured with BMAs (31). Unlike the pro-tumor effect of leptin, most of the studies demonstrated that adiponectin had anti-tumor effects (83). The MMTV-PyVT spontaneous mammary tumor mouse model with reduced adiponectin expressions facilitated breast tumor formation by downregulating mitogen-activated protein kinase (MAPK) 3 and phosphatase and tensin homolog deleted on chromosome 10 (PTEN) expression and activating PI3K/Akt signaling (84, 85) and the phosphorylation of ERK 1/2 (86). As for colon cancer, adiponectin inhibited the proliferation of tumor cells by blocking the cell division cycle at G1/S boundary. This suppressive effect was attenuated by knockdown of AdipoR1 and AdipoR2 in colon cancer cells (87). These experiments indicate that the integrity of adiponectin/AdipoR1/2 signaling is indispensable for anti-tumor effects, including anti-tumorigenesis and anti-proliferation effects. Adiponectin exerts the anti-proliferation effect partly via AMPK phosphorylation (87, 88), which is the central regulator of energy balance and many other signaling pathways. On the other hand, the pro-apoptotic effect of adiponectin is mediated by enhancing JNK phosphorylation and reducing mTOR phosphorylation in liver cancer (89). Despite substantial evidence of its anti-metastatic effect, a small number of studies showed that adiponectin could promote the tumor growth and migration (90, 91). Adiponectin could promote ceramide activity and the production of the anti-apoptotic substance—phingosine-1-phosphate (S1P) (92). Amazingly, S1P transporter spinster homolog 2 (Spns2), which promotes the secretion of S1P, was recently identified as a key modulator that facilitated tumor metastasis from 810 rodent cancer cell lines through *in vivo* experiments (93). The different results of adiponectin in tumor metastasis may derive from the multiple isoforms of AdipoR (91).

Recently, *in vivo* experiences raise the possibility that adiponectin may be effective to prevent or treat bone metastasis. MM is characterized by multiple osteolytic lesions, which is similar to osteolytic damages in bone metastasis. Fowler et al. administrated an apolipoprotein mimetic peptide (L-4F) to enhance the circulation adiponectin in MM model. They found that L-4F administration led to remarkably fewer osteolytic lesions and tumor burden and more bone formation (94). More importantly, the effects of L-4F appear to require adiponectin, for there was no apparent effect in adiponectin-deficient mice.

### The mutual relationship between leptin and adiponectin in tumor growth and metastasis

The opposite effects between leptin and adiponectin in bone marrow environment make their mutual relationship in bone metastasis more complicated. In general, if the beneficial effects of adiponectin are overwhelmed by the adverse effects of leptin, metabolic disorders may appear. This was confirmed in clinical experiments and animal models. Imbalance of leptin/adiponectin (L/A) ratio was regarded as the biomarker of metabolism diseases, including obesity and diabetes (95–98). However, the consequences of the increased ratio of L/A derived from BMAs are not fully understood. Higher L/A ratio was associated with endometrial cancer aggressiveness (99). An *in vitro* experiment showed that adiponectin administration attenuated leptin-induced accelerated tumor growth of hepatic carcinoma cells and mammary tumor cells MCF-7 (85, 89). On the other hand, leptin treatment inhibited the anti-proliferation effect and pro-apoptosis effect (Bcl-2) of adiponectin in prostate cancer cell line (86, 100). An *in vivo* experiment confirmed the mutual relationship between leptin and adiponectin. Simultaneously using adiponectin together with leptin inhibited the anti-proliferation effect of adiponectin in the liver cancer model (89).

Which one, leptin or adiponectin, may play a decisive role in bone metastasis in the bone marrow microenvironment? Two main elements can be used to analyze this issue. First of all, which adipokine is more abundant in bone marrow? Both leptin and adiponectin exert dose-related effects on tumor cells. Thus, higher concentration may seize advantage. The concentration of adiponectin in human serum ranged from 2 to 20 μg/ml (101). In contrast, leptin had a larger fluctuation and much lower scope, ranging from 5 to 20 ng/ml in non-overweight people, but reaching to 50 ng/ml or greater in overweight and obesity individuals (86). Compared with WAT, lower expression of leptin was identified in BMAs using gene chip and qRT-PCR in mouse (27), but the primary culture of human BMAs showed the opposite results (28). A recent clinical study found that BMAs in breast cancer patients but not healthy controls were positively associated with serum leptin level. Thus, it seems that leptin expression could be enhanced in some human cancers. Another study, which explored the relationship between leptin and adiponectin in breast cancer patients, is consistent with this assumption. Chen and colleagues found that breast cancer patients had higher serum leptin concentration but lower serum adiponectin concentration, thus leading to a significant higher L/A ratio than control group (102). More intriguingly, L/A ratio but not serum levels of leptin or adiponectin alone, was positively associated with tumor size. It seems that L/A ratio determines the final consequence in tumor growth in the bone marrow niche. However, the concentration of leptin and adiponectin in the bone marrow is unknown. We can only speculate that L/A ratio is also increased in the bone marrow microenvironment. The increased ratio of L/A could promote the colonization and growth of tumor cells in the bone marrow niche. Second, which receptors are more abundant in tumor cells? The mRNA level of LepR, AdipoR1 and AdipoR2 is low in normal mouse prostate (103), but the situation in the prostate cancer is different. Both AdipR1/2 and LepR are expressed in prostate tumor tissues and prostate tumor cell lines, but the distribution is entirely different. AdipoR1 and AdipoR2 are mainly located on the apical sites of epithelial cells whereas LepR can be widely detected through tumors (86). In addition, LepR was highly expressed in breast cancer (104), lung cancer (105) and colorectal carcinoma (106). High expression of LepR was associated with high advanced stage. Instead, decreased AdipoR1/R2 expression was found in lung cancer (107) and colorectal cancer patients (108). The positive rate of staining against AdipoR1 and AdipoR2 in human breast cancer tissue was only 30.4% and 26.3%, separately (109). In summary, LepR may dominate in many kinds of tumor tissues. The increased L/A ratio and wide distribution of LepR could create a suitable niche for tumor growth in the bone marrow. In summary, the endocrine functions of BMAs could provide assistance for tumor progression and bone metastasis.

## Inflammatory factors of BMAs on bone metastasis

### The different abundance of interleukin-6 (IL-6) and tumor necrosis factor-α (TNF-α) in WAT and BMAs

Chronic inflammation state in obesity patients is associated with increased risk of cancer (110). IL-6 and TNF-α are two main proinflammatory cytokines that are significantly increased in obesity patients with excessive WAT (111). But the main source of IL-6 and TNF-α in obesity patients is not only from WAT. More strikingly, Stuart and colleagues found that almost all TNF-α and significant amount of IL-6 were derived from macrophages that infiltrated in adipose tissues but not adipocytes themselves (112). This study has a hint that adipocytes may release different amount of TNF-α and IL-6. Besides, Caja and Puerta also found the different abundance of IL-6 and TNF-α in the visceral and subcutaneous adipose tissues, with IL-6 ranging from 80 to 500 pg/100 mg tissues and TNF-α fewer than 10 pg/100 mg tissues (113). An *in vitro* experiment using cytokine array showed the high level of IL-6 in human adipocyte-conditioned media (114). An *in vivo* human experiment brought into a similar conclusion. The production of IL-6 from the subcutaneous WAT made up an estimated 15–25% of the systemic IL-6 at midday and 25–35% at night; however, the secretion of TNF-α from the subcutaneous WAT was uncertain (115). In co-culture condition, although both IL-6 and TNF-α mRNA level in adipocytes were significantly upregulated in the presence of breast tumor cells, the level of TNF-α was undetectable in the supernatant of adipocytes with or without breast tumor cells. By contrast, IL-6 was remarkably higher in the supernatant (48). The abundance of IL-6 and the deficiency of TNF-α in WAT were also confirmed in BMAs. Human BMAs also released abundant amount of IL-6 *in vitro*, but only subtle level of TNF-α could be detected (28). More remarkably, BMAs produced more amount of IL-6 than WAT (22). Upregulation of IL-6 could be beneficial for tumor progression and bone metastasis.

### The pro-tumor effects of IL-6 in tumor microenvironment

IL-6 is overexpressed in tumor microenvironment, in which tumor surrounding adipocytes are referred as cancer-associated adipocytes (48). Previous studies showed that IL-6 from adipose stromal cells (ASCs) promoted the migration and invasion of ER (-) mammary cancer (116). Interestingly, a recent study found that stromal IL-6 was actually released by adipocytes adjacent to cancer cells (48). Presumably, IL-6 secreted from BMAs may have the same functions as ASCs. IL-6 exerts its effect by binding to IL-6 receptor (IL6R). With the increasing level of IL-6 produced by adipocytes, IL-6R was upregulated in EpCAMþ/CD133þ cancer stem cells. In this co-culture system, c-Met, STAT3 and ERK1/2 signaling pathways were activated, whereas Wingless-related integration site (Wnt)/β-catenin signaling was unchanged (114). BMA-derived IL-6 plays an indispensable role in tumor metastasis. The mRNA expression of IL-6 was upregulated by 3-fold in breast tumor cells when co-cultured with adipocytes, whereas both IL-1β and TNFα were not obviously affected (117). Adipocyte-derived IL-6 attracted ovarian cancer cells to a large fat pad omentum (36). Binding of IL-6 to its receptor activates at least two signaling pathways in the IL-6 expressing cancer cells. Firstly, activation of Jak2/STAT-3 signaling leads to the induction of epithelial-mesenchymal transition (EMT), accompanied by the upregulation of E-cadherin, N-cadherin, MMP-7 and MMP-9 (118) and the enhanced metastatic potential of cancer cells (119). Secondly, IL-6 could activate PI3K/Akt pathway and thus promote cancer cell survival (120, 121). Even if the bone-invasive tumor cells did not express IL6R, BMAs may still be able to induce these signaling cascades. Soluble IL6R (sIL6R), is a special form of IL6R that could bind the ligand IL-6 and activate a process named trans-signaling (122). Clinical research showed that adipocyte size was positively correlated with sIL6R independent of body mass index (BMI) (123). In patients with knee osteoarthritis, sIL6R could be detected in both subcutaneous WAT and the infrapatellar fat pad. The release of sIL6R in the infrapatellar fat pad was 3.6-fold higher than that in subcutaneous WAT (124). We speculate that a larger amount of sIL6R in infrapatellar fat pad may be associated with the local inflammatory microenvironment. Considering the abundant immune cells and inflammatory factors in the bone marrow microenvironment, BMAs may also have the potential to release a significant amount of sIL6R and bind with IL6 to activate the trans-signaling in tumor cells. What is worse, IL-6 itself could increase the numbers of BMAs, thus creating a positive feedback (125). Antibodies of IL-6 would significantly inhibit the pro-metastasis effect of adipocytes when co-cultured with breast tumor cells (48). IL-6 neutralization significantly blocked the seeding and metastatic growth of breast cancer cells in the bone (126). In addition, tocilizumab, a human monoclonal antibody against IL-6R, could reduce serum receptor activator of nuclear factor kappa B ligand (RANKL) levels and suppress skeletal tumor growth in prostate cancer model (127). This indicates that IL-6 could be a potential target for the prevention and treatment of bone metastasis.

In addition to IL-6, adipocytes could secrete other cytokines, including IL-1α (50), IL-1β (116), IL-8 (36, 114), IL-15, IL-16 (22), chemokine (C-X-C motif) ligand 1(CXCL1) and CXCL2 (50). The above-mentioned cytokines have been found to enhance the development of bone metastasis.

## Integrating BMAs into the old “vicious cycle”

The old “vicious cycle” is traditionally used to illustrate the association among tumor cells, osteoblasts and osteoclasts. In this cycle, tumor cells release multiple factors to enhance the osteolytic destruction, followed by the release of growth factors (TGF-β, etc.) from bone matrix, thus creating a “vicious cycle” (128). However, the “vicious cycle” in the bone microenvironment is far more complicated than that. Regarding the abundance and the significant role of BMAs and their precursors, they should be integrated into the old “vicious cycle.”

As we know, bone-forming osteoblasts and bone-resorbing osteoclasts precisely coordinate to maintain the dynamic balance of bone metabolism. RANKL and macrophage colony-stimulating factor (M-CSF) are two indispensable factors for osteoclast differentiation as well as bone resorption (129). M-CSF and RANKL are released by osteoblasts/stromal cells to enhance the osteoclast formation. However, recent studies indicated that M-CSF and RANKL could be released by the very early stage of BMA precursors; what is more amazing, those two factors released by BMA precursors could induce the osteoclasts formation in the co-culture condition (130). But once BMA precursors were differentiated into mature BMAs, the expression of RANKL and M-CSF was reduced significantly (130, 131). Another study may explain the reason of this phenomenon. They found that the early adipogenesis marker C/EBPβ and C/EBPδ but not the late adipogenesis marker PPARγ, could bind to the promoter of RANKL and promote its expression (132). Thus, it is speculated that mature BMAs may only release a medium amount of RANKL. More notably, RANKL was only released by BMAs but not WAT (125). This indicates that BMAs could directly regulate bone remodeling and bone destruction.

PTHrP is produced by many tumor cells and is well-known for its contribution to hypercalcaemia (133). PTHrP could bind to parathyroid hormone (PTH)/PTHrP receptor (PTH1R) and activate multiple signaling transmissions within cells. More interesting, PTHrP could only enhance bone metastasis, but not visceral metastasis (lung, liver, kidney and lymph node) (134).The role of PTHrP in tumor survival and invasion is closely related with BMAs. On one hand, PTHrP could downregulate the expression of PPARγ, and thus adipogenic differentiation is inhibited (135). As we know, adipogenesis is a process of enhanced uptake of glucose followed by *de novo* lipogenesis in adipocytes. Perhaps this process consumes large amounts of glucose. Therefore, tumor cells release PTHrP to inhibit adipogenesis and compete for the limited nutrition (mainly glucose) in the bone marrow. On the other hand, PTHrP could upregulate the thermogenic gene expression of mature adipocytes (including uncoupling protein 1 (UCP1) and iodothyronine deiodinase 2 (Dio2) (by 200-and 20-fold, separately). This led to lipolysis of mature adipocytes (136). Thus, tumor cells may obtain energy from the mature adipocytes in this way. Recent studies revealed the association of PTHrP and RANKL. Interestingly, PTH1R knockout in BMAs resulted in much higher expression of RANKL, BMSCs in the absence of PTH1R were preferentially differentiated into mature BMAs. It seems that blocking of PTHrP/PTH1R signaling could promote adipogenesis and increase the expression of RANKL in the bone marrow (125). We speculate that PTHrP and RANKL could promote tumor growth in bone independently.

## BMAs could be the potential target for bone metastasis

As stated above, BMAs are the potential target to conquer bone metastasis, thus it may be necessary to reduce BMA formation to inhibit bone metastasis. Recent studies tried to explore the methods to reduce or get rid of BMAs. Considering the specificity of adiponectin for adipocytes, Adiponectin-Cre/ER (*Adipoq-*Cre/ER), which can recombine in BMAs, was therefore used to eliminate BMAs. Adipoq-cre/ER; R26^iDTR^; R26^tdTomato^ transgenic mice were injected with tamoxifen and subsequently diphtheria toxin (57). This method did initially ablate almost all BMAs after treatment with diphtheria toxin for 3 days. Surprisingly, rapid regeneration of BMAs was observed after 14 days. These adipocytes may derive from unrecombined BMA progenitors. Thus, it is not an effective method to get rid of BMAs. As we know, C/EBPα and PPARγ are two indispensable factors in adipocyte differentiation. Thus, they could be the primary targets to regulate adipocyte formation including WAT and BMAs. Global PPARγ knockout mice were not able to survive (137). Similarly, C/EBPα knockout mice died soon after birth with no visible WAT (138). Considering the important roles of C/EBPα and PPARγ in other important physiological functions, Moitra and colleagues established the viable “fatless” A-ZIP/F1 transgenic mice. A-ZIP/F is a designed dominant-negative protein that blocks the DNA combination of both C/EBP and Jun families. These “fatless” mice had no WAT throughout lifespan. However, these mice still had small amount of BMAs in the femurs and tibias (57). More interestingly, BMAs in A-ZIP/F1 transgenic mice increased significantly after irradiations. These results indicate that, BMAs are more difficult to eliminate than WAT. The precursor cells of BMAs may be more complex and is tough to totally eliminate.

In humans, congenital generalized lipodystrophy (CGL), which is an autosomal recessive disease, shed light on the research of BMAs. Different gene mutations have been identified in four subtypes of CGL: 1-acylglycerol-3-phosphate O-acyltransferase 2 (AGPAT2), Berardinelli-Seip congenital lipodystrophy 2 (BSCL2 or seipin), caveolin 1 (CAV1) and polymerase I and transcript release factor (PTRF) mutation are responsible for CGL1, CGL2, CGL3, and CGL4, respectively (139). Despite the nearly complete lack of peripheral WAT in patients with CGL, BMAs are well preserved in patients with CAV1 (CGL3) or PTRF (CGL4) mutation. Both Cav1 and Ptrf knockout led to obviously reduced amount of WAT in mouse genetic models. However, their influences on BMAs are different. BMAs were well preserved in Cav1 knockout mice; however, Ptrf knockout resulted in a significant reduction of rBMAs (proximal tibial) instead of cBMAs (distal tibia) (9). This indicates that CAV1 may only influence WAT whereas PTRF could influence both WAT and rBMAs. The latter could be more important for bone metastasis. In this way, PTRF could be the potential target for the prevention and treatment of bone metastasis. Additionally, the lack of BMAs was observed in both CGL1 and CGL2 patients. Compared with AGPAT2 gene mutation in CGL1 patients, CGL2 patients with seipin mutations exhibited significantly severe lack of WAT and BMAs (140). Recent studies also confirmed its important roles in fat store in both drosophila and human (141). It seems that seipin mutations have a far greater impact on BMAs than other genes.

PTH could be used to inhibit BMA formation. PTH (1–34) significantly inhibited the adipogenic differentiation of human BMSCs *in vitro* (142). Daily injection of 25nmol/kg PTH (1–34) in mice remarkably reduced BMAs in proximal epiphyses and the growth plate near the tibial fibular junction, which could be classified as rBMAs. Coincidentally, the proximal tibia was much easier to occur bone metastasis than the tibial diaphysis and distal end in human (143). The number of BMAs was significantly reduced in patients treated with PTH (1–34) for 18 months (125). It seems that PTH (1–34) could inhibit the rBMAs formation, which is more important in bone metastasis. However, the results from different *in vivo* experiments involving PTH were strikingly different. Schneider et al. showed that skeletal metastasis to hind limb metaphyses and the craniofacial region was 3-fold higher in intermittent PTH-treated prostate cancer-bearing mice (144). By contrasts, Swami et al. demonstrated that intermittent PTH administration could significantly reduce the frequency of spontaneous metastasis to hind limbs and increase bone volume in breast cancer model (145). Actually, they used the same dose of PTH (80 μg/kg/d) but the duration of the treatment was considerably different (2 weeks vs. 8 weeks). Another reason that may explain this discrepancy is that the effect of PTH (1–34) on bone metastasis may be largely dependent on tumor types. Previous studies have showed that high serum PTH was common in prostate cancer patients and was inversely associated with mortality (146).

Sclerostin (SOST) is secreted by osteocytes and functions as a Wnt signaling inhibitory molecule. In addition to its inhibitory effects on osteoblast differentiation and bone formation, sclerostin is a newly identified factor that regulates the formation and metabolism of adipocytes (147, 148). Sost knockout mice exhibited a significant reduction in the whole-body fat mass and adipocyte size. Similarly, Sost-neutralizing antibody reduced the accumulation of WAT (148). Consistent with the above results, genetic ablation and pharmaceutical inhibition of Sost led to a significantly decrease in the number and size of BMAs in the proximal and distal end of the long bone (149). Interestingly, strongly positive expression of Sost was detected in tumor tissues from breast cancer patients with bone metastasis; Sost antibody remarkably inhibited bone metastasis and osteolytic destruction in breast cancer model (150). Recently, romosozumab, a monoclonal antibody to Sost, has been used successfully in treating osteoporosis (151). This suggests that it is possible to use Sost antibody to reduce BMAs and treat bone metastasis. However, opposite results were found in a prostate cancer model. Hudson et al. demonstrated that overexpressing Sost in prostate cancer cell line (PC-3) remarkably suppressed the metastasis and osteolytic destruction (152). In summary, Targeting BMA for the prevention and treatment of bone metastasis is promising but not yet practical.

## Conclusion

There are still much unknown in BMAs and bone metastasis. Accumulating evidence supports the pro-tumor roles of BMA in skeleton metastasis. One distinguished feature of BMAs in bone metastasis is that they could be the energy source of tumor cells. So far, no other cells except adipocytes could directly provide FFA as energy source for adjacent tumor cells. The blocking of this process may help to inhibit bone metastasis. In addition, the endocrine functions of BMAs have two sides in bone metastasis. IL-6 and other inflammatory factors have doubtless pro-tumor effects. However, leptin and adiponectin have the opposite effects on bone metastasis. Other adipocytokines could also influence bone metastasis. The complexity of BMA function in different conditions may contribute to different results in bone metastasis. Considering the abundance of BMAs in bone marrow, the regulation of BMA formation and endocrine functions could be a new avenue to treat bone metastasis. Destruction of the suitable microenvironment created by BMAs may help to inhibit bone metastasis. It is an emerging direction that is totally different from previous treatment options. Destroying the “soil” but not directly killing the “seed” may be a new strategy to prevent and treat bone metastasis.

## Author contributions

XY and YH designed this review. GL, XY, and YH wrote the manuscript.

### Conflict of interest statement

The authors declare that the research was conducted in the absence of any commercial or financial relationships that could be construed as a potential conflict of interest.

## References

[1] Esposito M Guise T Kang Y. The biology of bone metastasis. Cold Spring Harb Perspect Med. (2017) 8:a031252. 10.1101/cshperspect.a031252 29101110 PMC5980796

[2] Rucci N Teti A. Osteomimicry: how the seed grows in the soil. Calcif Tissue Int. (2017) 102:131–40. 10.1007/s00223-017-0365-1 29147721

[3] Luo G He Y Zhao Q Yu X. Immune cells act as promising targets for the treatment of bone metastasis. Recent Pat Anticancer Drug Discov. (2017) 12:221–33. 10.2174/1574892812666170606123113 28595538

[4] Fidler IJ. Metastasis: quantitative analysis of distribution and fate of tumor emboli labeled with 125 I-5-iodo-2'-deoxyuridine. J Natl Cancer Inst. (1970) 45:773–82. 5513503

[5] Fazeli PK Horowitz MC MacDougald OA Scheller EL Rodeheffer MS Rosen CJ. Marrow fat and bone–new perspectives. J Clin Endocrinol Metab. (2013) 98:935–45. 10.1210/jc.2012-3634 23393168 PMC3590487

[6] Veldhuis-Vlug AG Rosen CJ. Clinical implications of bone marrow adiposity. J Intern Med. (2017) 283:121–39. 10.1111/joim.12718 29211319 PMC5847297

[7] Cawthorn WP Scheller EL Learman BS Parlee SD Simon BR Mori H. Bone marrow adipose tissue is an endocrine organ that contributes to increased circulating adiponectin during caloric restriction. Cell Metab. (2014) 20:368–75. 10.1016/j.cmet.2014.06.003 24998914 PMC4126847

[8] Boumelhem BB Assinder SJ Bell-Anderson KS Fraser ST. Flow cytometric single cell analysis reveals heterogeneity between adipose depots. Adipocyte (2017) 6:112–23. 10.1080/21623945.2017.1319536 28453382 PMC5477740

[9] Scheller EL Doucette CR Learman BS Cawthorn WP Khandaker S Schell B. Region-specific variation in the properties of skeletal adipocytes reveals regulated and constitutive marrow adipose tissues. Nat Commun. (2015) 6:7808. 10.1038/ncomms8808 26245716 PMC4530473

[10] Afshin A Forouzanfar MH Reitsma MB Sur P Estep K Lee A. Health effects of overweight and obesity in 195 Countries over 25 years. N Engl J Med. (2017) 377:13–27. 10.1056/NEJMoa1614362 28604169 PMC5477817

[11] Steele CB Thomas CC Henley SJ Massetti GM Galuska DA Agurs-Collins T. Vital signs: trends in incidence of cancers associated with overweight and obesity - United States, 2005-2014. MMWR Morb Mortal Wkly Rep. (2017) 66:1052–8. 10.15585/mmwr.mm6639e1 28981482 PMC5720881

[12] Veld J O'Donnell EK Reagan MR Yee AJ Torriani M Rosen CJ. Abdominal adipose tissue in MGUS and multiple myeloma. Skeletal Radiol. (2016) 45:1277–83. 10.1007/s00256-016-2425-4 27344672

[13] Langley RR Fidler IJ. The seed and soil hypothesis revisited–the role of tumor-stroma interactions inmetastasis to different organs. Int J Cancer (2011) 128:2527–35. 10.1002/ijc.26031 21365651 PMC3075088

[14] Mado K Ishii Y Mazaki T Ushio M Masuda H Takayama T. A case of bone metastasis of colon cancer that markedly responded to S-1/CPT-11 combination chemotherapy and became curable by resection. World J Surg Oncol. (2006) 4:3. 10.1186/1477-7819-4-3 16417646 PMC1395313

[15] Roth ES Fetzer DT Barron BJ Joseph UA Gayed IW Wan DQ. Does colon cancer ever metastasize to bone first? a temporal analysis of colorectal cancer progression. BMC Cancer (2009) 9:274. 10.1186/1471-2407-9-274 19664211 PMC2734866

[16] Yang L Drake BF Colditz GA. Obesity and other cancers. J Clin Oncol. (2016) 34:4231–37. 10.1200/JCO.2016.68.4837 27903157

[17] Tewari R Rajender S Natu SM Goel A Dalela D Goel MM. Significance of obesity markers and adipocytokines in high grade and high stage prostate cancer in North Indian men - a cross-sectional study. Cytokine (2013) 63:130–34. 10.1016/j.cyto.2013.04.008 23669251

[18] Vidal AC Howard LE Moreira DM Castro-Santamaria R Andriole GJ Freedland SJ. Obesity increases the risk for high-grade prostate cancer: results from the REDUCE study. Cancer Epidemiol Biomarkers Prev. (2014) 23:2936–42. 10.1158/1055-9965.EPI-14-0795 25261967 PMC4257871

[19] Kyrgiou M Kalliala I Markozannes G Gunter MJ Paraskevaidis E Gabra H. Adiposity and cancer at major anatomical sites: umbrella review of the literature. BMJ (2017) 356:j477. 10.1136/bmj.j477 28246088 PMC5421437

[20] Smith L Brinton LA Spitz MR Lam TK Park Y Hollenbeck AR. Body mass index and risk of lung cancer among never, former, and current smokers. J Natl Cancer Inst. (2012) 104:778–89. 10.1093/jnci/djs179 22457475 PMC3352831

[21] Pulido C Vendrell I Ferreira AR Casimiro S Mansinho A Alho I. Bone metastasis risk factors in breast cancer. Ecancermedicalscience (2017) 11:715. 10.3332/ecancer.2017.715 28194227 PMC5295847

[22] Majka SM Fox KE Psilas JC Helm KM Childs CR Acosta AS. De novo generation of white adipocytes from the myeloid lineage via mesenchymal intermediates is age, adipose depot, and gender specific. Proc Natl Acad Sci USA. (2010) 107:14781–6. 10.1073/pnas.1003512107 20679227 PMC2930432

[23] Ambrosi TH Scialdone A Graja A Gohlke S Jank AM Bocian C. Adipocyte accumulation in the bone marrow during obesity and aging impairs stem cell-based hematopoietic and bone regeneration. Cell Stem Cell (2017) 20:771–84. 10.1016/j.stem.2017.02.009 28330582 PMC5459794

[24] Yue R Zhou BO Shimada IS Zhao Z Morrison SJ. Leptin receptor promotes adipogenesis and reduces osteogenesis by regulating mesenchymal stromal cells in adult bone marrow. Cell Stem Cell (2016) 18:782–96. 10.1016/j.stem.2016.02.015 27053299

[25] O'Halloran N Courtney D Kerin MJ Lowery AJ. Adipose-derived stem cells in novel approaches to breast reconstruction: their suitability for tissue engineering and oncological safety. Breast Cancer (2017) 11:1178223417726777. 10.1177/1178223417726777 29104428 PMC5562338

[26] Tang W Zeve D Suh JM Bosnakovski D Kyba M Hammer RE. White fat progenitor cells reside in the adipose vasculature. Science (2008) 322:583–6. 10.1126/science.1156232 18801968 PMC2597101

[27] Liu LF Shen WJ Ueno M Patel S Kraemer FB. Characterization of age-related gene expression profiling in bone marrow and epididymal adipocytes. BMC Genomics (2011) 12:212. 10.1186/1471-2164-12-212 21545734 PMC3113784

[28] Laharrague P Fontanilles AM Tkaczuk J Corberand JX Penicaud L Casteilla L. Inflammatory/haematopoietic cytokine production by human bone marrow adipocytes. Eur Cytokine Netw. (2000) 11:634–9. 11125307

[29] Trotter TN Gibson JT Sherpa TL Gowda PS Peker D Yang Y. Adipocyte-lineage cells support growth and dissemination of multiple myeloma in bone. Am J Pathol. (2016) 186:3054–63. 10.1016/j.ajpath.2016.07.012 27648615 PMC5222958

[30] Falank C Fairfield H Reagan MR. Signaling interplay between bone marrow adipose tissue and multiple myeloma cells. Front Endocrinol. (2016) 7:67. 10.3389/fendo.2016.00067 27379019 PMC4911365

[31] Tabe Y Yamamoto S Saitoh K Sekihara K Monma N Ikeo K. Bone marrow adipocytes facilitate fatty acid oxidation activating AMPK and a transcriptional network supporting survival of acute monocytic leukemia cells. Cancer Res. (2017) 77:1453–64. 10.1158/0008-5472.CAN-16-1645 28108519 PMC5354955

[32] Arner P Langin D. Lipolysis in lipid turnover, cancer cachexia, and obesity-induced insulin resistance. Trends Endocrinol Metab. (2014) 25:255–62. 10.1016/j.tem.2014.03.002 24731595

[33] Chowdhury R Amin MA Bhattacharyya K. Intermittent fluorescence oscillations in lipid droplets in a live normal and lung cancer cell: time-resolved confocal microscopy. J Phys Chem B (2015) 119:10868–75. 10.1021/jp5120042 25674799

[34] Abramczyk H Surmacki J Kopec M Olejnik AK Lubecka-Pietruszewska K Fabianowska-Majewska K. The role of lipid droplets and adipocytes in cancer. Raman imaging of cell cultures: MCF10A, MCF7, and MDA-MB-231 compared to adipocytes in cancerous humanbreast tissue. Analyst (2015) 140:2224–35. 10.1039/c4an01875c 25730442

[35] Fairfield H Falank C Farrell M Vary C Boucher JM Driscoll H. Development of a 3D bone marrow adipose tissue model. Bone (2018). [Epub ahead of print]. 10.1016/j.bone.2018.01.023 29366838 PMC6062483

[36] Nieman KM Kenny HA Penicka CV Ladanyi A Buell-Gutbrod R Zillhardt MR. Adipocytes promote ovarian cancer metastasis and provide energy for rapid tumor growth. Nat Med. (2011) 17:1498–503. 10.1038/nm.2492 22037646 PMC4157349

[37] Svensson RU Parker SJ Eichner LJ Kolar MJ Wallace M Brun SN. Inhibition of acetyl-CoA carboxylase suppresses fatty acid synthesis and tumor growth of non-small-cell lung cancer in preclinical models. Nat Med. (2016) 22:1108–19. 10.1038/nm.4181 27643638 PMC5053891

[38] Nelson ME Lahiri S Chow JD Byrne FL Hargett SR Breen DS. Inhibition of hepatic lipogenesis enhances liver tumorigenesis by increasing antioxidant defence and promoting cell survival. Nat Commun. (2017) 8:14689. 10.1038/ncomms14689 28290443 PMC5424065

[39] Pascual G Avgustinova A Mejetta S Martin M Castellanos A Attolini CS. Targeting metastasis-initiating cells through the fatty acid receptor CD36. Nature (2017) 541:41–5. 10.1038/nature20791 27974793

[40] Hovey RC Aimo L. Diverse and active roles for adipocytes during mammary gland growth and function. J Mammary Gland Biol Neoplasia (2010) 15:279–90. 10.1007/s10911-010-9187-8 20717712 PMC2941079

[41] Wen YA Xing X Harris JW Zaytseva YY Mitov MI Napier DL. Adipocytes activate mitochondrial fatty acid oxidation and autophagy to promote tumor growth in colon cancer. Cell Death Dis. (2017) 8:e2593. 10.1038/cddis.2017.21 28151470 PMC5386470

[42] Wang J Chen GL Cao S Zhao MC Liu YQ Chen XX. Adipogenic niches for melanoma cell colonization and growth in bone marrow. Lab Invest. (2017) 97:737–45. 10.1038/labinvest.2017.14 28218738

[43] Argiles JM Busquets S Stemmler B Lopez-Soriano FJ. Cancer cachexia: understanding the molecular basis. Nat Rev Cancer (2014) 14:754–62. 10.1038/nrc3829 25291291

[44] Das SK Eder S Schauer S Diwoky C Temmel H Guertl B. Adipose triglyceride lipase contributes to cancer-associated cachexia. Science (2011) 333:233–38. 10.1126/science.1198973 21680814

[45] Herroon MK Rajagurubandara E Hardaway AL Powell K Turchick A Feldmann D. Bone marrow adipocytes promote tumor growth in bone via FABP4-dependent mechanisms. Oncotarget (2013) 4:2108–23. 10.18632/oncotarget.1482 24240026 PMC3875773

[46] Shafat MS Oellerich T Mohr S Robinson SD Edwards DR Marlein CR. Leukemic blasts program bone marrow adipocytes to generate a pro-tumoral microenvironment. Blood (2017) 129:1320–32. 10.1182/blood-2016-08-734798 28049638

[47] Elliott BE Tam SP Dexter D Chen ZQ. Capacity of adipose tissue to promote growth and metastasis of a murine mammary carcinoma: effect of estrogen and progesterone. Int J Cancer (1992) 51:416–24. 1317363 10.1002/ijc.2910510314

[48] Dirat B Bochet L Dabek M Daviaud D Dauvillier S Majed B. Cancer-associated adipocytes exhibit an activated phenotype and contribute to breast cancer invasion. Cancer Res. (2011) 71:2455–65. 10.1158/0008-5472.CAN-10-3323 21459803

[49] Miranda F Mannion D Liu S Zheng Y Mangala LS Redondo C. Salt-inducible kinase 2 couples ovarian cancer cell metabolism with survival at the adipocyte-rich metastatic niche. Cancer Cell (2016) 30:273–89. 10.1016/j.ccell.2016.06.020 27478041

[50] Ye H Adane B Khan N Sullivan T Minhajuddin M Gasparetto M. Leukemic stem cells evade chemotherapy by metabolic adaptation to an adipose tissue niche. Cell Stem Cell (2016) 19:23–37. 10.1016/j.stem.2016.06.001 27374788 PMC4938766

[51] Hui SK Arentsen L Sueblinvong T Brown K Bolan P Ghebre RG. A phase I feasibility study of multi-modality imaging assessing rapid expansion of marrow fat and decreased bone mineral density in cancer patients. Bone (2015) 73:90–7. 10.1016/j.bone.2014.12.014 25536285 PMC4336831

[52] Justesen J Stenderup K Ebbesen EN Mosekilde L Steiniche T Kassem M. Adipocyte tissue volume in bone marrow is increased with aging and in patients with osteoporosis. Biogerontology (2001) 2:165–71. 10.1023/A:1011513223894 11708718

[53] Behan JW Yun JP Proektor MP Ehsanipour EA Arutyunyan A Moses AS. Adipocytes impair leukemia treatment in mice. Cancer Res. (2009) 69:7867–74. 10.1158/0008-5472.CAN-09-0800 19773440 PMC2756308

[54] Liu Z Xu J He J Liu H Lin P Wan X. Mature adipocytes in bone marrow protect myeloma cells against chemotherapy through autophagy activation. Oncotarget (2015) 6:34329–41. 10.18632/oncotarget.6020 26455377 PMC4741456

[55] Perera RJ Marcusson EG Koo S Kang X Kim Y White N. Identification of novel PPARgamma target genes in primary human adipocytes. Gene (2006) 369:90–9. 10.1016/j.gene.2005.10.021 16380219

[56] Furuhashi M Hotamisligil GS. Fatty acid-binding proteins: role in metabolic diseases and potential as drug targets. Nat Rev Drug Discov. (2008) 7:489–503. 10.1038/nrd2589 18511927 PMC2821027

[57] Zhou BO Yu H Yue R Zhao Z Rios JJ Naveiras O. Bone marrow adipocytes promote the regeneration of stem cells and haematopoiesisby secreting SCF. Nat Cell Biol. (2017) 19:891–903. 10.1038/ncb3570 28714970 PMC5536858

[58] Bajaj MS Kulkarni RS Ghode SS Limaye LS Kale VP. Irradiation-induced secretion of BMP4 by marrow cells causes marrow adipogenesispost-myelosuppression. Stem Cell Res. (2016) 17:646–53. 10.1016/j.scr.2016.11.015 27865162

[59] Chi M Chen J Ye Y Tseng HY Lai F Tay KH. Adipocytes contribute to resistance of human melanoma cells to chemotherapy and targeted therapy. Curr Med Chem. (2014) 21:1255–67. 10.2174/0929867321666131129114742 24304284

[60] Yu W Cao DD Li QB Mei HL Hu Y Guo T. Adipocytes secreted leptin is a pro-tumor factor for survival of multiple myeloma under chemotherapy. Oncotarget (2016) 7:86075–86. 10.18632/oncotarget.13342 27863383 PMC5349898

[61] Boyd AL Reid JC Salci KR Aslostovar L Benoit YD Shapovalova Z. Acute myeloid leukaemia disrupts endogenous myelo-erythropoiesis by compromisingthe adipocyte bone marrow niche. Nat Cell Biol. (2017) 19:1336–47. 10.1038/ncb3625 29035359

[62] Laharrague P Larrouy D Fontanilles AM Truel N Campfield A Tenenbaum R. High expression of leptin by human bone marrow adipocytes in primary culture. FASEB J. (1998) 12:747–52. 9619453 10.1096/fasebj.12.9.747

[63] Chen XX Yang T. Roles of leptin in bone metabolism and bone diseases. J Bone Miner Metab. (2015) 33:474–85. 10.1007/s00774-014-0569-7 25777984

[64] Zhou BO Yue R Murphy MM Peyer JG Morrison SJ. Leptin-receptor-expressing mesenchymal stromal cells represent the main source of bone formed by adult bone marrow. Cell Stem Cell (2014) 15:154–68. 10.1016/j.stem.2014.06.008 24953181 PMC4127103

[65] Templeton ZS Lie WR Wang W Rosenberg-Hasson Y Alluri RV Tamaresis JS. Breast cancer cell colonization of the human bone marrow adipose tissue niche. Neoplasia (2015) 17:849–61. 10.1016/j.neo.2015.11.005 26696367 PMC4688564

[66] Snoussi K Strosberg AD Bouaouina N Ben AS Helal AN Chouchane L. Leptin and leptin receptor polymorphisms are associated with increased risk and poor prognosis of breast carcinoma. BMC Cancer (2006) 6:38. 10.1186/1471-2407-6-38 16504019 PMC1397853

[67] Howard JM Pidgeon GP Reynolds JV. Leptin and gastro-intestinal malignancies. Obes Rev. (2010) 11:863–74. 10.1111/j.1467-789X.2010.00718.x 20149119

[68] Jarde T Perrier S Vasson MP Caldefie-Chezet F. Molecular mechanisms of leptin and adiponectin in breast cancer. Eur J Cancer (2011) 47:33–43. 10.1016/j.ejca.2010.09.005 20889333

[69] Kumar J Fang H McCulloch DR Crowley T Ward AC. Leptin receptor signaling via Janus kinase 2/Signal transducer and activator of transcription 3 impacts on ovarian cancer cell phenotypes. Oncotarget (2017) 8:93530–40. 10.18632/oncotarget.19873 29212170 PMC5706816

[70] Cleary MP Juneja SC Phillips FC Hu X Grande JP Maihle NJ Leptin receptor-deficient MMTV-TGF-alpha/Lepr(db)Lepr(db) female mice do not develop oncogene-induced mammary tumors. Exp Biol Med. (2004) 229:182–93. 10.1177/153537020422900207 14734797

[71] Feng H Liu Q Zhang N Zheng L Sang M Feng J. Leptin promotes metastasis by inducing an epithelial-mesenchymal transition in A549 lung cancer cells. Oncol Res. (2013) 21:165–71. 10.3727/096504014X13887748696662 24512731

[72] Samuel-Mendelsohn S Inbar M Weiss-Messer E Niv-Spector L Gertler A Barkey RJ. Leptin signaling and apoptotic effects in human prostate cancer cell lines. Prostate (2011) 71:929–45. 10.1002/pros.21309 21541970

[73] Omoto Y Iwase H. Clinical significance of estrogen receptor beta in breast and prostate cancer from biological aspects. Cancer Sci. (2015) 106:337–43. 10.1111/cas.12613 25611678 PMC4409875

[74] Zhang Y Daquinag A Traktuev DO Amaya-Manzanares F Simmons PJ March KL. White adipose tissue cells are recruited by experimental tumors and promote cancer progression in mouse models. Cancer Res. (2009) 69:5259–66. 10.1158/0008-5472.CAN-08-3444 19491274 PMC3857703

[75] Deo DD Rao AP Bose SS Ouhtit A Baliga SB Rao SA. Differential effects of leptin on the invasive potential of androgen-dependent and -independent prostate carcinoma cells. J Biomed Biotechnol. (2008) 2008:163902. 10.1155/2008/163902 18584049 PMC2435597

[76] Onuma M Bub JD Rummel TL Iwamoto Y. Prostate cancer cell-adipocyte interaction: leptin mediates androgen-independentprostate cancer cell proliferation through c-Jun NH2-terminal kinase. J Biol Chem. (2003) 278:42660–7. 10.1074/jbc.M304984200 12902351

[77] Hoda MR Theil G Mohammed N Fischer K Fornara P. The adipocyte-derived hormone leptin has proliferative actions on androgen-resistant prostate cancer cells linking obesity to advanced stages of prostate cancer. J Oncol. (2012) 2012:280386. 10.1155/2012/280386 22690216 PMC3368429

[78] Sakaguchi S Goto H Hanibuchi M Otsuka S Ogino H Kakiuchi S. Gender difference in bone metastasis of human small cell lung cancer, SBC-5 cells in natural killer-cell depleted severe combined immunodeficient mice. Clin Exp Metastasis (2010) 27:351–9. 10.1007/s10585-010-9333-0 20464627

[79] Martin J Arm J Smart J Palazzi K Capp A Ainsworth P. Spinal multiparametric MRI and DEXA changes over time in men with prostate cancer treated with androgen deprivation therapy: a potential imaging biomarker of treatment toxicity. Eur Radiol. (2017) 27:995–1003. 10.1007/s00330-016-4434-z 27287481

[80] Fruebis J Tsao TS Javorschi S Ebbets-Reed D Erickson MR Yen FT. Proteolytic cleavage product of 30-kDa adipocyte complement-related protein increases fatty acid oxidation in muscle and causes weight loss in mice. Proc Natl Acad Sci USA. (2001) 98:2005–10. 10.1073/pnas.041591798 11172066 PMC29372

[81] Yamauchi T Kamon J Ito Y Tsuchida A Yokomizo T Kita S. Cloning of adiponectin receptors that mediate antidiabetic metabolic effects. Nature (2003) 423:762–9. 10.1038/nature01705 12802337

[82] Beltowski J. Adiponectin and resistin–new hormones of white adipose tissue. Med Sci Monit. (2003) 9:A55–61. 12601307

[83] Dalamaga M Diakopoulos KN Mantzoros CS. The role of adiponectin in cancer: a review of current evidence. Endocr Rev. (2012) 33:547–94. 10.1210/er.2011-1015 22547160 PMC3410224

[84] Lam JB Chow KH Xu A Lam KS Liu J Wong NS. Adiponectin haploinsufficiency promotes mammary tumor development in MMTV-PyVT mice by modulation of phosphatase and tensin homolog activities. PLoS ONE (2009) 4:e4968. 10.1371/journal.pone.0004968 19319191 PMC2656613

[85] Jarde T Caldefie-Chezet F Goncalves-Mendes N Mishellany F Buechler C Penault-Llorca F. Involvement of adiponectin and leptin in breast cancer: clinical and in vitro studies. Endocr Relat Cancer (2009) 16:1197–210. 10.1677/ERC-09-0043 19661131

[86] Grossmann ME Mizuno NK Bonorden MJ Ray A Sokolchik I Narasimhan ML. Role of the adiponectin leptin ratio in prostate cancer. Oncol Res. (2009) 18:269–77. 10.3727/096504009X12596189659367 20225764

[87] Kim AY Lee YS Kim KH Lee JH Lee HK Jang SH. Adiponectin represses colon cancer cell proliferation via AdipoR1- and -R2-mediated AMPK activation. Mol Endocrinol. (2010) 24:1441–52. 10.1210/me.2009-0498 20444885 PMC5417469

[88] Fogarty S Hardie DG. Development of protein kinase activators: AMPK as a target in metabolic disorders and cancer. Biochim Biophys Acta (2010) 1804:581–91. 10.1016/j.bbapap.2009.09.012 19778642

[89] Sharma D Wang J Fu PP Sharma S Nagalingam A Mells J. Adiponectin antagonizes the oncogenic actions of leptin in hepatocellular carcinogenesis. Hepatology (2010) 52:1713–22. 10.1002/hep.23892 20941777 PMC2967627

[90] Jia Z Liu Y Cui S. Adiponectin induces breast cancer cell migration and growth factor expression. Cell Biochem Biophys. (2014) 70:1239–45. 10.1007/s12013-014-0047-9 24906235

[91] Libby EF Frost AR Demark-Wahnefried W Hurst DR. Linking adiponectin and autophagy in the regulation of breast cancer metastasis. J Mol Med. (2014) 92:1015–23. 10.1007/s00109-014-1179-5 24903246 PMC4197061

[92] Holland WL Miller RA Wang ZV Sun K Barth BM Bui HH. Receptor-mediated activation of ceramidase activity initiates the pleiotropic actions of adiponectin. Nat Med. (2011) 17:55–63. 10.1038/nm.2277 21186369 PMC3134999

[93] Weyden LV Arends MJ Campbell AD Bald T Wardle-Jones H Griggs N. Genome-wide in vivo screen identifies novel host regulators of metastatic colonization. Nature (2017) 541:233–6. 10.1038/nature20792 28052056 PMC5603286

[94] Fowler JA Lwin ST Drake MT Edwards JR Kyle RA Mundy GR. Host-derived adiponectin is tumor-suppressive and a novel therapeutic target formultiple myeloma and the associated bone disease. Blood (2011) 118:5872–82. 10.1182/blood-2011-01-330407 21908434 PMC3228502

[95] Li G Xu L Zhao Y Li L Fu J Zhang Q. Leptin-adiponectin imbalance as a marker of metabolic syndrome among Chinese children and adolescents: the BCAMS study. PLoS ONE (2017) 12:e186222. 10.1371/journal.pone.0186222 29020116 PMC5636141

[96] Lopez-Jaramillo P Gomez-Arbelaez D Lopez-Lopez J Lopez-Lopez C Martinez-Ortega J Gomez-Rodriguez A. The role of leptin/adiponectin ratio in metabolic syndrome and diabetes. Horm Mol Biol Clin Invest. (2014) 18:37–45. 10.1515/hmbci-2013-0053 25389999

[97] Ayina C Endomba F Mandengue SH Noubiap J Ngoa L Boudou P. Association of the leptin-to-adiponectin ratio with metabolic syndrome in a sub-Saharan African population. Diabetol Metab Syndr. (2017) 9:66. 10.1186/s13098-017-0265-6 28878827 PMC5584041

[98] Zhang M Cheng H Zhao X Hou D Yan Y Cianflone K. Leptin and leptin-to-adiponectin ratio predict adiposity gain in nonobese children over a six-year period. Child Obes. (2017) 13:213–21. 10.1089/chi.2016.0273 28128972 PMC5444411

[99] Ashizawa N Yahata T Quan J Adachi S Yoshihara K Tanaka K. Serum leptin-adiponectin ratio and endometrial cancer risk in postmenopausal female subjects. Gynecol Oncol. (2010) 119:65–9. 10.1016/j.ygyno.2010.07.007 20674961

[100] Mistry T Digby JE Desai KM Randeva HS. Leptin and adiponectin interact in the regulation of prostate cancer cell growthvia modulation of p53 and bcl-2 expression. BJU Int. (2008) 101:1317–22. 10.1111/j.1464-410X.2008.07512.x 18279445

[101] Arita Y Kihara S Ouchi N Takahashi M Maeda K Miyagawa J. Paradoxical decrease of an adipose-specific protein, adiponectin, in obesity. 1999. Biochem Biophys Res Commun. (2012) 425:560–4. 10.1016/j.bbrc.2012.08.024 22925674

[102] Chen DC Chung YF Yeh YT Chaung HC Kuo FC Fu OY. Serum adiponectin and leptin levels in Taiwanese breast cancer patients. Cancer Lett. (2006) 237:109–14. 10.1016/j.canlet.2005.05.047 16019138

[103] Sarmento-Cabral A L-Lopez F Luque RM. Adipokines and their receptors are widely expressed and distinctly regulated by the metabolic environment in the prostate of male mice: direct role under normaland tumoral conditions. Endocrinology (2017) 158:3540–52. 10.1210/en.2017-00370 28938461

[104] Artac M Altundag K. Leptin and breast cancer: an overview. Med Oncol. (2012) 29:1510–4. 10.1007/s12032-011-0056-0 21877194

[105] Xu YJ Shao YF Zhao X Geng YT Wang K Yin YM. Expression and clinical significance of leptin, the functional receptor of leptin (OB-Rb) and HER-2 in non-small-cell lung cancer: a retrospective analysis. J Cancer Res Clin Oncol. (2011) 137:1841–8. 10.1007/s00432-011-1054-5 21927908 PMC11827768

[106] Liu H Wan D Pan Z Cao L Wu X Lu Z. Expression and biological significance of leptin, leptin receptor, VEGF, and CD34 in colorectal carcinoma. Cell Biochem Biophys. (2011) 60:241–4. 10.1007/s12013-010-9145-5 21161731

[107] Abdul-Ghafar J Oh SS Park SM Wairagu P Lee SN Jeong Y. Expression of adiponectin receptor 1 is indicative of favorable prognosis in non-small cell lung carcinoma. Tohoku J Exp Med. (2013) 229:153–62. 10.1620/tjem.229.153 23358237

[108] Ayyildiz T Dolar E Ugras N Adim SB Yerci O. Association of adiponectin receptor (Adipo-R1/-R2) expression and colorectal cancer. Asian Pac J Cancer Prev. (2014) 15:9385–90. 25422229 10.7314/apjcp.2014.15.21.9385

[109] Korner A Pazaitou-Panayiotou K Kelesidis T Kelesidis I Williams CJ Kaprara A. Total and high-molecular-weight adiponectin in breast cancer: in vitro and in vivo studies. J Clin Endocrinol Metab. (2007) 92:1041–8. 10.1210/jc.2006-1858 17192291

[110] Iyengar NM Gucalp A Dannenberg AJ Hudis CA. Obesity and cancer mechanisms: tumor microenvironment and inflammation. J Clin Oncol. (2016) 34:4270–6. 10.1200/JCO.2016.67.4283 27903155 PMC5562428

[111] Jorge AS Andrade JM Paraiso AF Jorge GC Silveira CM de Souza LR. Body mass index and the visceral adipose tissue expression of IL-6 and TNF-alphaare associated with the morphological severity of non-alcoholic fatty liver disease in individuals with class III obesity. Obes Res Clin Pract. (2016) 12:1–8. 10.1016/j.orcp.2016.03.009 27083404

[112] Weisberg SP McCann D Desai M Rosenbaum M Leibel RL Ferrante AJ. Obesity is associated with macrophage accumulation in adipose tissue. J Clin Invest. (2003) 112:1796–808. 10.1172/JCI19246 14679176 PMC296995

[113] Caja S Puerta M. White adipose tissue production and release of IL-6 and TNF-alpha do not parallel circulating and cerebrospinal fluid concentrations in pregnant rats. Horm Metab Res. (2008) 40:375–80. 10.1055/s-2008-1062701 18401835

[114] Firtina KZ Koc D Sahin E Avci ST Yilmaz M Atabey N. Effect of adipocyte-secreted factors on EpCAM+/CD133+ hepatic stem cell population. Biochem Biophys Res Commun. (2016) 474:482–90. 10.1016/j.bbrc.2016.04.137 27131739

[115] Mohamed-Ali V Goodrick S Rawesh A Katz DR Miles JM Yudkin JS. Subcutaneous adipose tissue releases interleukin-6, but not tumor necrosis factor-alpha, in vivo. J Clin Endocrinol Metab. (1997) 82:4196–200. 10.1210/jcem.82.12.4450 9398739

[116] Walter M Liang S Ghosh S Hornsby PJ Li R. Interleukin 6 secreted from adipose stromal cells promotes migration and invasion of breast cancer cells. Oncogene (2009) 28:2745–55. 10.1038/onc.2009.130 19483720 PMC2806057

[117] Bochet L Meulle A Imbert S Salles B Valet P Muller C. Cancer-associated adipocytes promotes breast tumor radioresistance. Biochem Biophys Res Commun. (2011) 411:102–6. 10.1016/j.bbrc.2011.06.101 21712027

[118] Kong G Jiang Y Sun X Cao Z Zhang G Zhao Z. Irisin reverses the IL-6 induced epithelial-mesenchymal transition in osteosarcoma cell migration and invasion through the STAT3/Snail signaling pathway. Oncol Rep. (2017) 38:2647–56. 10.3892/or.2017.5973 29048621 PMC5780017

[119] Wang L Cao L Wang H Liu B Zhang Q Meng Z. Cancer-associated fibroblasts enhance metastatic potential of lung cancer cells through IL-6/STAT3 signaling pathway. Oncotarget (2017) 8:76116–28. 10.18632/oncotarget.18814 29100297 PMC5652691

[120] Zhang J Choi Y Mavromatis B Lichtenstein A Li W. Preferential killing of PTEN-null myelomas by PI3K inhibitors through Akt pathway. Oncogene (2003) 22:6289–95. 10.1038/sj.onc.1206718 13679867

[121] Tu Y Gardner A Lichtenstein A. The phosphatidylinositol 3-kinase/AKT kinase pathway in multiple myeloma plasma cells: roles in cytokine-dependent survival and proliferative responses. Cancer Res. (2000) 60:6763–70. 11118064

[122] Knupfer H Preiss R. sIL-6R: more than an agonist? Immunol Cell Biol. (2008) 86:87–91. 10.1038/sj.icb.7100113 17724457

[123] Kuo FC Huang YH Lin FH Hung YJ Hsieh CH Lu CH. Circulating soluble IL-6 receptor concentration and visceral adipocyte size are related to insulin resistance in Taiwanese adults with morbid obesity. Metab Syndr Relat Disord. (2017) 15:187–93. 10.1089/met.2016.0135 28346858

[124] Distel E Cadoudal T Durant S Poignard A Chevalier X Benelli C. The infrapatellar fat pad in knee osteoarthritis: an important source of interleukin-6 and its soluble receptor. Arthritis Rheum. (2009) 60:3374–7. 10.1002/art.24881 19877065

[125] Fan Y Hanai JI Le PT Bi R Maridas D DeMambro V. Parathyroid hormone directs bone marrow mesenchymal cell fate. Cell Metab. (2017) 25:661–72. 10.1016/j.cmet.2017.01.001 28162969 PMC5342925

[126] Luo X Fu Y Loza AJ Murali B Leahy KM Ruhland MK. Stromal-initiated changes in the bone promote metastatic niche development. Cell Rep. (2016) 14:82–92. 10.1016/j.celrep.2015.12.016 26725121 PMC4706805

[127] Zheng Y Basel D Chow SO Fong-Yee C Kim S Buttgereit F. Targeting IL-6 and RANKL signaling inhibits prostate cancer growth in bone. Clin Exp Metastasis (2014) 31:921–33. 10.1007/s10585-014-9680-3 25223386

[128] Cook LM Shay G Araujo A Lynch CC. Integrating new discoveries into the “vicious cycle” paradigm of prostate to bone metastases. Cancer Metastasis Rev. (2014) 33:511–25. 10.1007/s10555-014-9494-4 24414228 PMC4096318

[129] Okamoto K Nakashima T Shinohara M Negishi-Koga T Komatsu N Terashima A. Osteoimmunology: the conceptual framework unifying the immune and skeletal systems. Physiol Rev. (2017) 97:1295–349. 10.1152/physrev.00036.2016 28814613

[130] Holt V Caplan AI Haynesworth SE. Identification of a subpopulation of marrow MSC-derived medullary adipocytes that express osteoclast-regulating molecules: marrow adipocytes express osteoclast mediators. PLoS ONE (2014) 9:e108920. 10.1371/journal.pone.0108920 25302610 PMC4193782

[131] Lanotte M Metcalf D Dexter TM. Production of monocyte/macrophage colony-stimulating factor by preadipocyte celllines derived from murine marrow stroma. J Cell Physiol. (1982) 112:123–7. 10.1002/jcp.1041120118 6980887

[132] Takeshita S Fumoto T Naoe Y Ikeda K. Age-related marrow adipogenesis is linked to increased expression of RANKL. J Biol Chem. (2014) 289:16699–710. 10.1074/jbc.M114.547919 24753250 PMC4059115

[133] Sen S Dasgupta P Kamath G Srikanth HS. Paratharmone related protein (peptide:A novel prognostic, diagnostic and therapeutic marker in Head &amp; Neck cancer. J Stomatol Oral Maxillofac Surg. (2017) 119:33–6. 10.1016/j.jormas.2017.10.016 29081380

[134] Miki T Yano S Hanibuchi M Kanematsu T Muguruma H Sone S. Parathyroid hormone-related protein (PTHrP) is responsible for production of bone metastasis, but not visceral metastasis, by human small cell lung cancer SBC-5 cells in natural killer cell-depleted SCID mice. Int J Cancer (2004) 108:511–5. 10.1002/ijc.11586 14696114

[135] Chan GK Deckelbaum RA Bolivar I Goltzman D Karaplis AC. PTHrP inhibits adipocyte differentiation by down-regulating PPAR gamma activity via a MAPK-dependent pathway. Endocrinology (2001) 142:4900–9. 10.1210/endo.142.11.8515 11606458

[136] Kir S White JP Kleiner S Kazak L Cohen P Baracos VE. Tumour-derived PTH-related protein triggers adipose tissue browning and cancer cachexia. Nature (2014) 513:100–4. 10.1038/nature13528 25043053 PMC4224962

[137] Barak Y Nelson MC Ong ES Jones YZ Ruiz-Lozano P Chien KR. PPAR gamma is required for placental, cardiac, and adipose tissue development. Mol Cell (1999) 4:585–95. 10549290 10.1016/s1097-2765(00)80209-9

[138] Wang ND Finegold MJ Bradley A Ou CN Abdelsayed SV Wilde MD. Impaired energy homeostasis in C/EBP alpha knockout mice. Science (1995) 269:1108–12. 7652557 10.1126/science.7652557

[139] Altay C Secil M Demir T Atik T Akinci G Ozdemir KN. Determining residual adipose tissue characteristics with MRI in patients with various subtypes of lipodystrophy. Diagn Interv Radiol. (2017) 23:428–34. 10.5152/dir.2017.17019 29044029 PMC5669542

[140] Simha V Garg A. Phenotypic heterogeneity in body fat distribution in patients with congenital generalized lipodystrophy caused by mutations in the AGPAT2 or seipin genes. J Clin Endocrinol Metab. (2003) 88:5433–7. 10.1210/jc.2003-030835 14602785

[141] Bi J Wang W Liu Z Huang X Jiang Q Liu G. Seipin promotes adipose tissue fat storage through the ER Ca(2)(+)-ATPase SERCA. Cell Metab. (2014) 19:861–71. 10.1016/j.cmet.2014.03.028 24807223

[142] Rickard DJ Wang FL Rodriguez-Rojas AM Wu Z Trice WJ Hoffman SJ. Intermittent treatment with parathyroid hormone (PTH) as well as a non-peptide small molecule agonist of the PTH1 receptor inhibits adipocyte differentiation in human bone marrow stromal cells. Bone (2006) 39:1361–72. 10.1016/j.bone.2006.06.010 16904389

[143] Greenbaum SL Thornhill BA Geller DS. Characterization and surgical management of metastatic disease of the Tibia. Am J Orthop. (2017) 46:E423–28. 29309457

[144] Schneider A Kalikin LM Mattos AC Keller ET Allen MJ Pienta KJ. Bone turnover mediates preferential localization of prostate cancer in the skeleton. Endocrinology (2005) 146:1727–36. 10.1210/en.2004-1211 15637291

[145] Swami S Johnson J Bettinson LA Kimura T Zhu H Albertelli MA. Prevention of breast cancer skeletal metastases with parathyroid hormone. JCI Insight (2017) 2:90874. 10.1172/jci.insight.90874 28878134 PMC5621896

[146] Schwartz GG. Prostate cancer, serum parathyroid hormone, and the progression of skeletal metastases. Cancer Epidemiol Biomarkers Prev. (2008) 17:478–83. 10.1158/1055-9965.EPI-07-2747 18349265

[147] Ukita M Yamaguchi T Ohata N Tamura M. Sclerostin Enhances Adipocyte Differentiation in 3T3-L1 Cells. J Cell Biochem. (2016) 117:1419–28. 10.1002/jcb.25432 26553151

[148] Kim SP Frey JL Li Z Kushwaha P Zoch ML Tomlinson RE. Sclerostin influences body composition by regulating catabolic and anabolic metabolism in adipocytes. Proc Natl Acad Sci USA. (2017) 114:E11238–47. 10.1073/pnas.1707876115 29229807 PMC5748171

[149] Fairfield H Falank C Harris E Demambro V McDonald M Pettitt JA. The skeletal cell-derived molecule sclerostin drives bone marrow adipogenesis. J Cell Physiol. (2018) 233:1156–67. 10.1002/jcp.25976 28460416 PMC5664178

[150] Zhu M Liu C Li S Zhang S Yao Q Song Q. Sclerostin induced tumor growth, bone metastasis and osteolysis in breast cancer. Sci Rep. (2017) 7:11399. 10.1038/s41598-017-11913-7 28900298 PMC5595999

[151] Saag KG Petersen J Brandi ML Karaplis AC Lorentzon M Thomas T. Romosozumab or alendronate for fracture prevention in women with osteoporosis. N Engl J Med. (2017) 377:1417–27. 10.1056/NEJMoa1708322 28892457

[152] Hudson BD Hum NR Thomas CB Kohlgruber A Sebastian A Collette NM. SOST inhibits prostate cancer invasion. PLoS ONE (2015) 10:e142058. 10.1371/journal.pone.0142058 26545120 PMC4636315

